# Supplementary material for: Mutation bias and GC content shape antimutator invasions
Source: Nat Commun. 2019 Jul 15;10:3114. doi: 10.1038/s41467-019-11217-6 (PMC6629674; doi:10.1038/s41467-019-11217-6)
Supplement: Supplementary file 1 — Supplementary Information [file 41467_2019_11217_MOESM1_ESM.pdf]

SUPPLEMENTARY INFORMATION

Mutation bias and GC content shape antimutator invasions

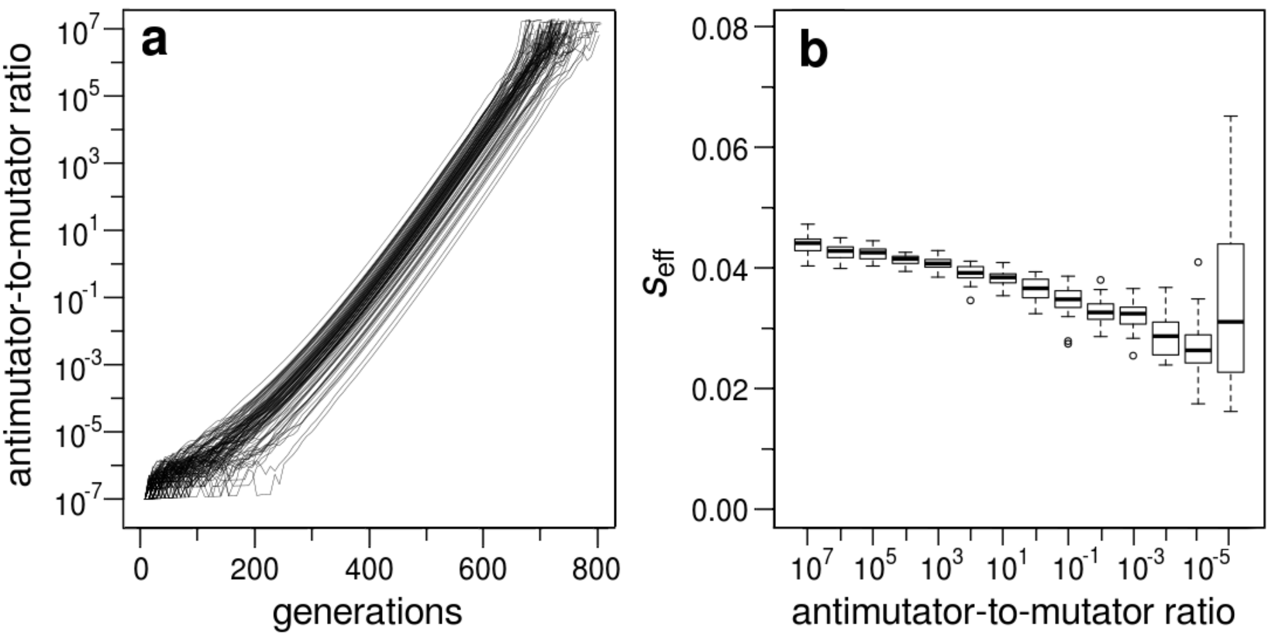

Supplementary Figure 1.- Estimating antimutator fitness. (a) Lines correspond to single antimutator alleles invading mutator populations. According to population genetics theory, the slope of these lines represent a good approximation of the selection coefficient of the invading allele[1]. The lines are curved, however, evidencing that the selection coefficient is not fixed, but increases with time. This is an unavoidable feature of trying to measure indirect selection, in which the advantage of the antimutator allele comes from its statistical association with a smaller load of deleterious mutations. Since the antimutator allele first arises on a mutator background, its advantage is initially non-existent, and it becomes manifest as mutations accumulate differentially over time. For this reason, we measured antimutator fitness as the average slope of the invasion curves over the entire fixation trajectory (see Methods) (b) Empirical estimates of the average effective selection coefficient (s_{eff}) at different points along the antimutator invasion trajectories depicted in (a) (that is, at different time points since the the allele is first introduced). Boxes and whiskers illustrate how the advantage of the antimutator allele is a function of the number of generations. All data comes from 100 independent simulations in which s_d =0.008 and m=300 (other parameters as described in Methods).

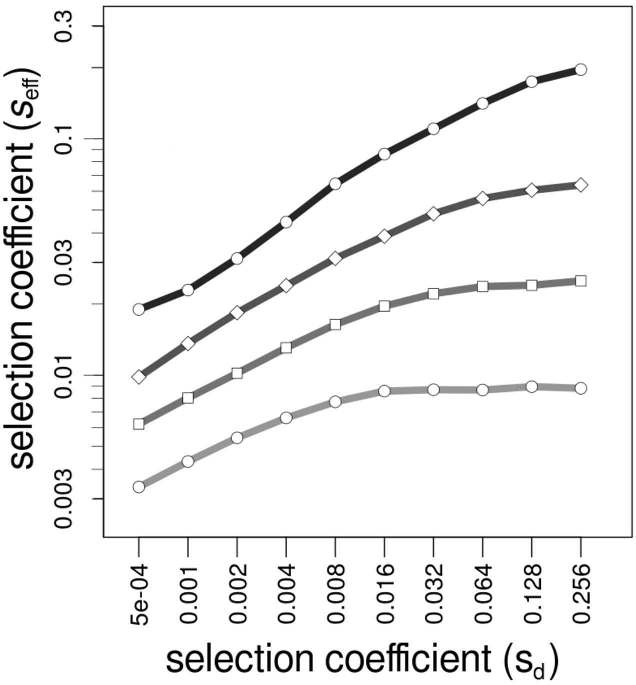

Supplementary Figure 2.- Dependence of antimutator dynamics on the fitness cost of deleterious mutations. Points represent the effective selection coefficient (s_{eff}) of invading antimutator alleles, averaged from 200 independent simulations. Lines depict different values of the mutation rate of the resident mutator (from top to bottom, *m = 1000*; b, *m = 300*; c, *m = 100*; d, *m = 30*). In line with the the Haldane-Muller principle, note how slopes become flatter as mutation rates are the smallest and fitness costs the highest. Other parameters as described in Methods.

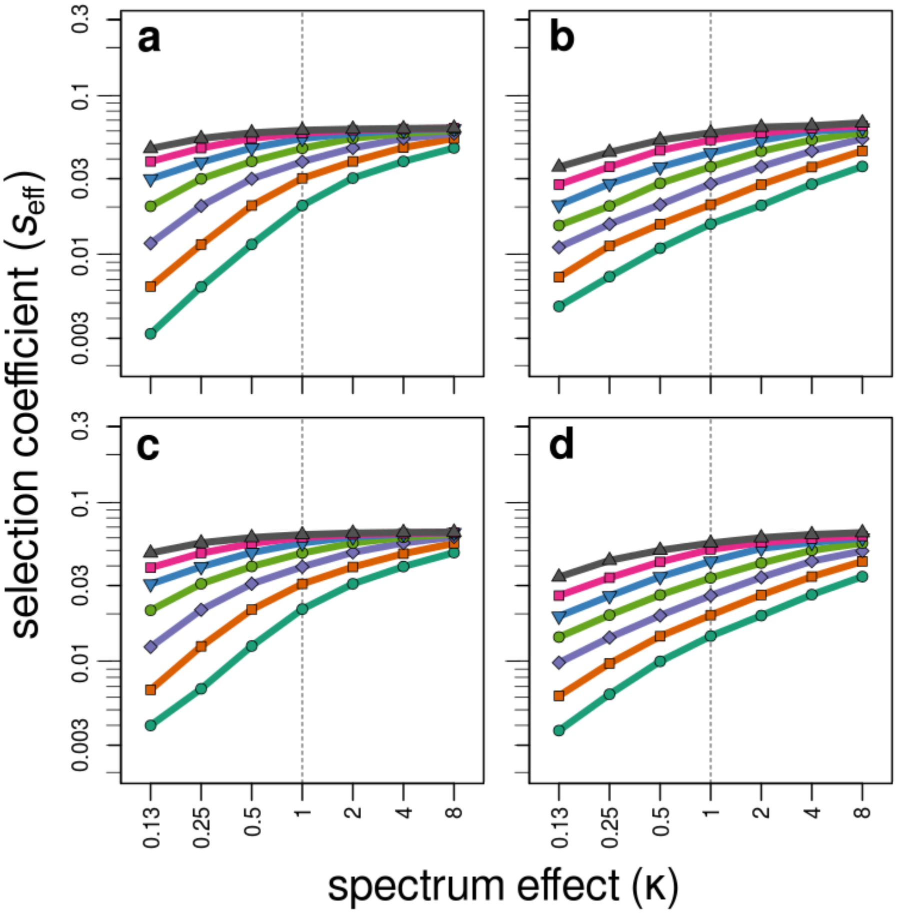

Supplementary Figure 3.- Impact of maximum population and bottleneck size on antimutator dynamics. Points and colours follow the same convention as in Figure 2. Panels (a) and (b) show antimutator fitness under two extreme values of the maximum population size (10^{11} and 10^5, respectively) while keeping the bottleneck size at 1/100, as in the LTEE. Panels (c) and (d) show antimutator fitness under two extreme values of the bottleneck size (1/2 and 1/10,000, respectively) while keeping the maximum population size at 10^9, as in the LTEE. Small population or bottleneck sizes have the effect of reducing s_{eff} over most combinations of κ and s_d. This is because the interplay between these two demographic parameters determines the minimum frequency at which any allele (mutator or antimutator) can be present in a population, and therefore how wide the range for the antimutator-to-mutator ratio can be. Narrow values for this range cause fixation times to be shorter, mutations to have less time to accumulate and, ultimately, effective selection coefficients to be smaller – as discussed in the caption of Figure S1. In all cases, the mutation rate of the resident mutator was fixed to $m=300$ (other parameters as described in Methods).

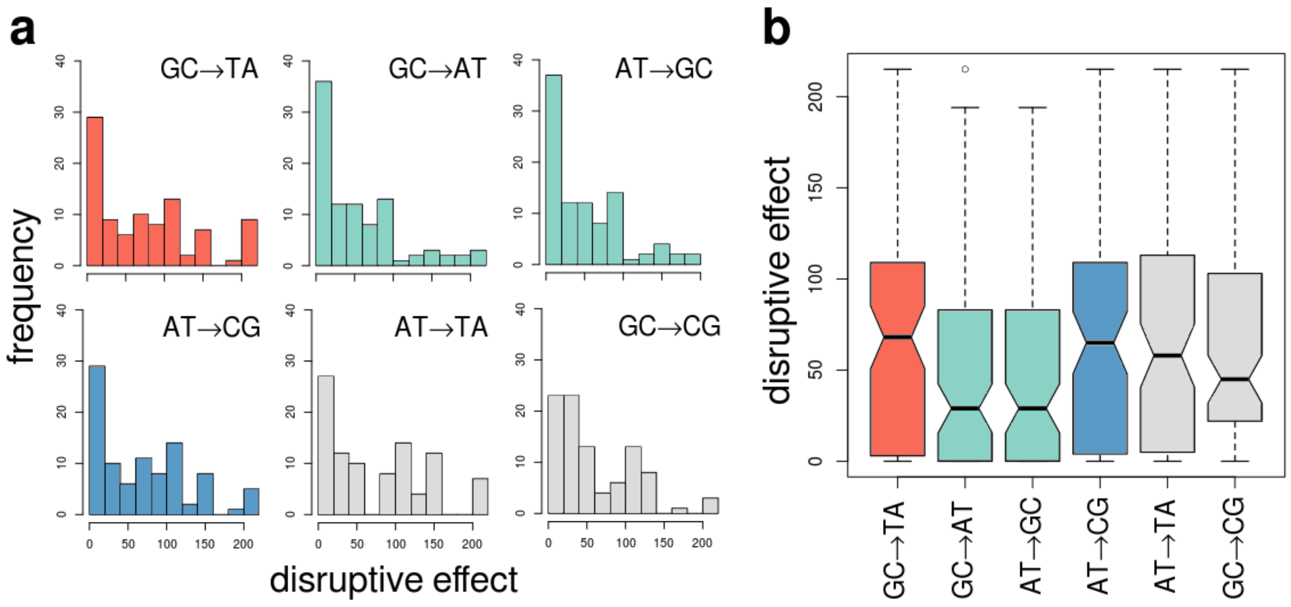

Supplementary Figure 4.- Protein-disrupting effects of mutations caused by all 6 possible base-substitution mutations across all codons (excluding stop codons). As in Figure 4, colours correspond to the specific mutations that match the mutational spectrum of *mutY*⁻ (red), *mutT*⁻ (blue) and Mismatch Repair⁻ (green) mutators. (a) Histograms showing the distribution of Grantham scores (b) Box-plots of the same scores. Note that, despite average differences, the distribution of scores display a large degree of overlap. Source data are provided as a Source Data file.

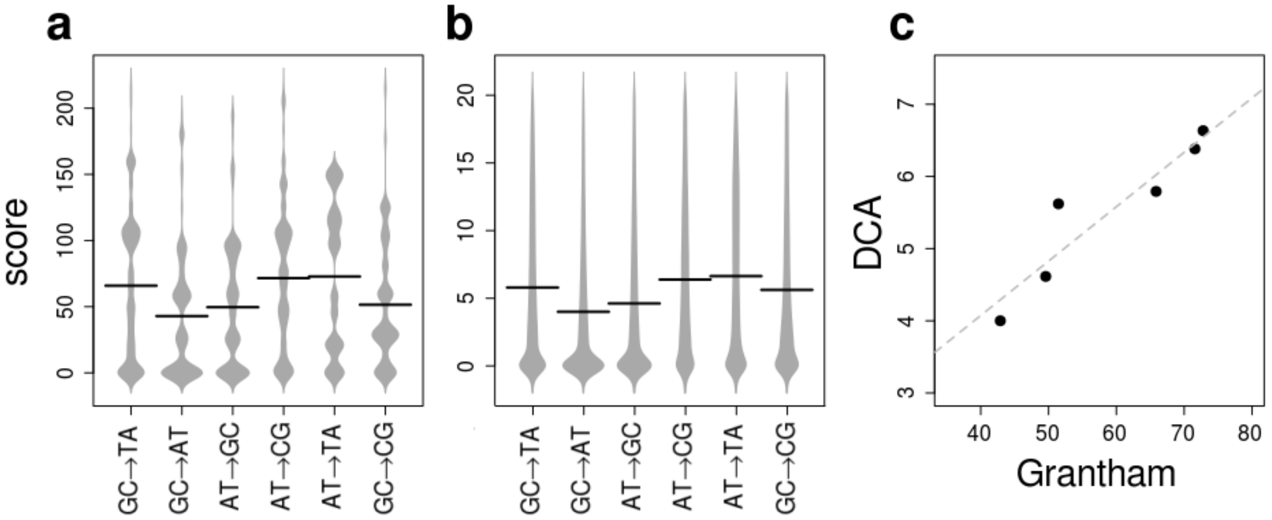

Supplementary Figure 5.- Comparison of Grantham's matrix with Direct Coupling Analysis (DCA) in predicting the fitness effects of non-synonymous mutations. Data comes from Couce *et al.* (2017)[2], in which both approaches were applied to genomes from the Lenski's Long-Term Evolution Experiment. Only deleterious mutations mapping to empirically validated essential or nearly-essential genes are included here. Panels (a) and (b) show the distribution of scores for Grantham's and DCA respectively. Panel (c) shows the correlation between both methods (*r =0.94*). Plots were drawn using the R library "beanplot"[3]. Source data are provided as a Source Data file.

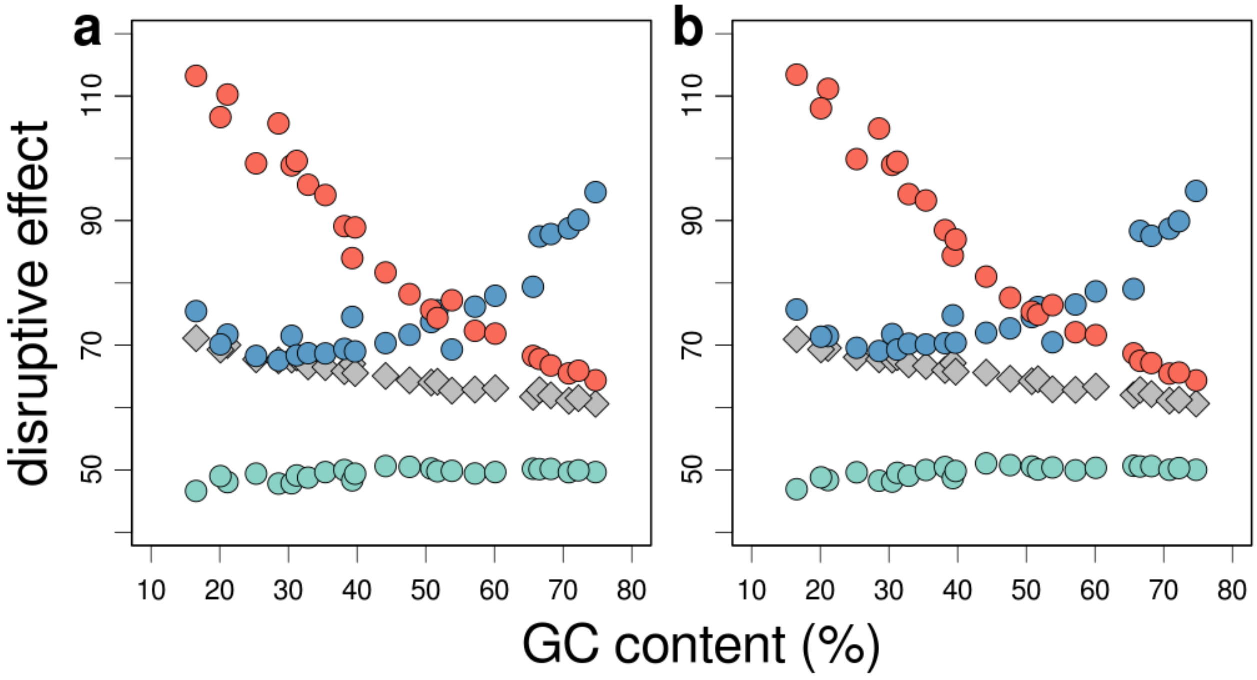

Supplementary Figure 6.- Average Grantham scores across a panel of species with genomes spanning a wide range of GC compositions. Panels correspond to analyses for genes belonging to the COG categories most commonly enriched in essential genes (H, J and M)[4] (a), and analyses for whole genomes (b). Colours and all other conventions are as in Figure 4. Source data are provided as a Source Data file.

Supplementary Table 1.- Bacterial genomes analyzed in this study

Species Name[1]	Gram	Taxonomy	%GC	Size (bp)	CDSs
Candidatus Carsonella ruddii PV	-	γ-proteobacteria	16.56	159662	184
Buchnera aphidicola Cc (Cinara cedri)	-	γ-proteobacteria	20.1	422434	378
Candidatus Sulcia muelleri CARI	-	Bacteroidetes	21.13	276511	266
Buchnera aphidicola Bp	-	γ-proteobacteria	25.3	615980	541
Clostridium difficile CD196	+	Firmicutes	28.56	4110554	3805
Campylobacter jejuni 4031	-	ϵ-proteobacteria	30.47	1669329	1711
Prochlorococcus marinus MIT 9312	-	Cyanobacteria	31.21	1709204	2027
Staphylococcus aureus subsp. aureus NCTC 8325	+	Firmicutes	32.87	2821361	2974
Bacillus anthracis Ames	+	Firmicutes	35.38	5227293	5844
Haemophilus influenzae Rd KW20	-	γ-proteobacteria	38.15	1830138	1821
Helicobacter pylori 2017	-	ϵ-proteobacteria	39.3	1548238	1682
Streptococcus pneumoniae D39	+	Firmicutes	39.71	2046115	2217
Xenorhabdus nematophila ATCC19061	-	γ-proteobacteria	44.15	4587917	4770
Yersinia pestis CO92	-	γ-proteobacteria	47.64	4829855	4587
Escherichia coli K12	-	γ-proteobacteria	50.79	4639675	4306
Neisseria meningitidis 053442	-	β-proteobacteria	51.7	2153416	2034
Corynebacterium glutamicum ATCC 13032	+	Actinobacteria	53.81	3309401	3223
Brucella abortus A13334	-	α-proteobacteria	57.22	3286032	3566
Bifidobacterium longum NCC2705	+	Actinobacteria	60.12	2260266	2049
Mycobacterium tuberculosis Beijing/NITR203	+	Actinobacteria	65.61	4411128	4699
Pseudomonas aeruginosa PAO1	-	γ-proteobacteria	66.56	6264404	5835
Burkholderia pseudomallei 1106b	-	β-proteobacteria	68.21	7224634	6990
Nocardia farcinica IFM 10152	+	Actinobacteria	70.83	6292344	6283
Streptomyces griseus subsp. griseus NBRC 13350	+	Actinobacteria	72.23	8545929	7547
Anaeromyxobacter dehalogenans 2CP-1	-	δ-proteobacteria	74.72	5029329	4690

[1] as retrieved from Genoscope (*www.genoscope.cns.fr*)

Supplementary References

1. Lenski, R. E., Quantifying fitness and gene stability in microorganisms. *Biotechnology* **15,** 173–192 (1991).
2. Couce, A. *et al.* Mutator genomes decay, despite sustained fitness gains, in a long-term experiment with bacteria. *Proc. Natl. Acad. Sci. U.S.A.* **114**, E9026–E9035 (2017).
3. Kampstra, P. Beanplot: A Boxplot Alternative for Visual Comparison of Distributions. *Journal of Statistical Software*, Code Snippets, **28** (2008).
4. Mandal, R. K., Jiang, T. & Kwon, Y. M. Essential genome of Campylobacter jejuni. *BMC Genomics* **18**, (2017).
